# Constitutive Phosphorylation of GATA-1 at Serine^26^ Attenuates the Colony-Forming Activity of Erythrocyte-Committed Progenitors

**DOI:** 10.1371/journal.pone.0064269

**Published:** 2013-05-22

**Authors:** Kou-Ray Lin, Chung-Leung Li, Jeffrey Jong-Young Yen, Hsin-Fang Yang-Yen

**Affiliations:** 1 Institute of Biomedical Sciences, Academia Sinica, Taipei, Taiwan; 2 Institute of Cell and Organismic Biology/Genomics Research Center, Academia Sinica, Taipei, Taiwan; 3 Institute of Molecular Biology, Academia Sinica, Taipei, Taiwan; French Blood Institute, France

## Abstract

We previously reported that IL-3 signaling induces phosphorylation of GATA-1 at the serine^26^ position, which contributes to IL-3-mediated anti-apoptotic response. Here, we demonstrate that phosphorylation of GATA-1 at serine^26^ is also transiently induced in cells of the erythroid lineage (primary erythroblasts and erythrocyte-committed progenitors [EPs]) by erythropoietin (EPO), the principal cytokine regulating erythropoiesis. To examine whether phosphorylation of GATA-1 at serine^26^ would have any impact on erythropoiesis, mutant mice carrying either a glutamic acid (GATA-1^S26E^) or alanine (GATA-1^S26A^) substitution at serine^26^ were generated. Neither GATA-1^S26E^ nor GATA-1^S26A^ mice showed any significant difference from control mice in peripheral blood cell composition under either steady state or stress conditions. The erythroblast differentiation in both mutant mice also appeared to be normal. However, a moderate reduction in the CFU-E progenitor population was consistently observed in the bone marrow of GATA-1^S26E^, but not GATA-1^S26A^ mice, suggesting that such defect was compensated for within the bone marrow. Surprisingly, reduced CFU-E progenitor population in GATA-1^S26E^ mice was mainly due to EPO-induced growth suppression of GATA-1^S26E^ EPs, albeit in the absence of EPO these cells manifested a survival advantage. Further analyses revealed that EPO-induced growth suppression of GATA-1^S26E^ EPs was largely due to the proliferation block resulted from GATA-1^S26E^-mediated transcriptional activation of the gene encoding the cell cycle inhibitor p21^Waf1/Cip1^. Taken together, these results suggest that EPO-induced transient phosphorylation of GATA-1 at serine^26^ is dispensable for erythropoiesis. However, failure to dephosphorylate this residue following its transient phosphorylation significantly attenuates the colony-forming activity of EPs.

## Introduction

Red blood cells differentiate from multi-potential hematopoietic stem cells in the bone marrow (BM) [1]. The committed progenitors are slowly proliferating erythroid burst-forming units (BFU-Es) [2]. These BFU-E cells divide and differentiate through the “mature” BFU-E stage into rapidly dividing erythroid colony-forming units (CFU-Es) [2], which differentiate further into erythroblasts, including the proerythroblast, basophilic, polychromatic, and orthochromatic erythroblast stages [3]. The nucleus shrinks and is shed as the erythroblast cells become reticulocytes before differentiating into erythrocytes [3]. As one of the most highly characterized differentiation model systems, erythrocyte differentiation has been shown to be regulated by many transcription factors, including the GATA family proteins [4], [5]. GATA-1 is the first identified member of the GATA family transcription factors [5], [6] that contains a transactivation domain in the N-terminal region and two zinc-finger domains for dimerization and DNA binding in the C-terminal region [7], [8], [9]. It is highly expressed in all erythroid cells [10], [11] and the functional GATA-binding DNA motif is present in the regulatory regions of virtually all erythroid-specific genes, including the globin gene family and genes coding for heme metabolism enzymes, erythroid transcription factors, and red cell membrane proteins [12], [13].

Phosphorylation has been suggested to affect GATA-1 function. MAPK-dependent phosphorylation has been shown to be involved in the regulation of GATA-1 protein stability [14]. It has also been reported that Akt-dependent phosphorylation of GATA-1 at serine^310^ is necessary for EPO-induced erythrocyte terminal differentiation in a proerythroblast cell line [15] and for EPO-induced TIMP1 secretion and maturation of fetal liver erythroid cells [16]. Surprisingly, in a knock-in animal model, it was shown that mutation of GATA-1 serine^310^ alone or triple mutations at serines 72, 142, and 310 did not significantly influence hematopoiesis *in vivo* [17].

In our previous study, we reported that MEK/MAPK-dependent phosphorylation of GATA-1 at serine^26^ was crucial for GATA-1 to mediate IL-3-dependent cell survival [18]. In an IL-3-dependent pro-B cell line, ectopic expression of mutant GATA-1 containing a glutamic acid substitution at residue 26, which mimics the negative charge of phospho-GATA-1, suppressed IL-3 deprivation-induced apoptosis and elevated the expression of endogenous anti-apoptotic protein Bcl-X_L_ [18]. In this study, we explored the possibility that phosphorylation of GATA-1 at serine^26^ might have some impacts on erythropoiesis, in which GATA-1 expression is essential [19]. We demonstrated that, in both primary erythroblasts and erythrocyte-committed progenitors (EPs), phosphorylation of GATA-1 at serine^26^ was transiently induced by EPO. By analyzing mutant mice harboring either a glutamic acid (GATA-1^S26E^) or alanine (GATA-1^S26A^) substitution at serine^26^, we demonstrate that phosphorylation of GATA-1 at serine^26^ is dispensable for erythropoiesis. However, failure to dephosphorylate this residue following its transient phosphorylation significantly attenuates the colony-forming activity of EPs. The underlying mechanism for the latter defect was also explored in this study.

## Materials and Methods

### Ethics Statement and Generation of GATA-1^S26E^ and GATA-1^S26A^ mice

To generate GATA-1 gene serine^26^ codon mutation alleles, two genomic fragments harboring the GATA-1 locus were isolated from a plasmid pSC3Z-mGATA-1 containing the 129/Svj mouse genomic DNA of interest (kindly provided by Dr. Sjaak Philipsen, Erasmus MC, Rotterdam, The Netherlands), and used to construct the targeting vectors. The 5′ fragments were engineered to contain either a glutamic acid (S26E) or alanine (S26A) codon at residue 26. These targeting vectors were constructed by PCR-assisted cloning so that a floxed cassette containing the Neo selection marker (neo^r^) was introduced into intron 2. Lying outside of the selection cassette were two homology arms (2.5 kb and 4 kb) and the gene encoding the thymidine kinase (TK) for negative selection. These targeting vectors were then electroporated into R1 embryonic stem (ES) cells, and Southern blotting using 5′ and 3′ probes external of the targeting construct was carried out to select clones that had undergone homologous recombination at the GATA-1 locus. Mutant ES cells were injected into C57BL/6 mouse blastocysts, and the resultant male chimeras were crossed with C57BL/6 females. To delete the neo^r^ cassette, GATA-1^S26E(neo+)^ and GATA-1^S26A(neo+)^ heterozygous females were further crossed with EIIa-Cre transgenic mice (originally on the FVB/N background, but backcrossed to C57BL/6 for more than seven generations; a gift from Dr. Ying-Hue Lee, IMB, Academia Sinica, Taipei, Taiwan). For some experiments, GATA-1^S26E^ mice were crossed with mutant mice carrying the null allele of the *Bim* or the *p21^Waf1/Cip1^* gene (purchased from The Jackson Laboratory) to generate compound mutant mice. Throughout this study, only male mice were used for all the analyses. Mice were housed under good animal care practice conditions and all experiments were performed with 8- to 9-weeks old males, under protocols approved by the Institutional Animal Care and Use Committee of the Academia Sinica.

### Analysis, Expansion and Purification of Primary Erythroblasts

To measure the percentage of erythroblasts at various developmental stages, total BM cells or splenocytes stained with FITC-labeled anti-CD71 (RI7217) and PE- labeled anti-Ter119 (TER-119) antibodies (BioLegend, San Diego, CA) were analyzed by flow cytometry using FACSCanto (BD Biosciences). Propidium iodide (PI) was added to the staining mixture to monitor cell viability. Purified anti-CD16/CD32 (FcγRIII/II) antibody (from 2.4G2 hybridoma supernatant) was also included in all analyses to block non-specific binding of anti-CD71 and anti-Ter119 antibodies to the FcγR on the cell surface. Primary erythroblasts were expanded and purified essentially as previously described [20]. Briefly, BM cells were harvested and cultured in StemPro-34 medium (Invitrogen) containing 2.5 U/mL of EPO (Amgen), 100 ng/mL of mouse stem cell factor (SCF), 7.5 µg/mL of insulin, 1 µM dexamethasone, 1 µM beta-estradiol, and 75 µg/mL of human transferrin (Sigma). Twenty-four hours after the culture was initiated, cells were supplemented with 0.5 volumes of fresh medium and cultured for another 24 hours before they were resuspended in 80% fresh medium plus 20% residual conditioned medium. Forty-eight hours later, cultured cells were stained with biotin-conjugated anti-CD71 (RI7217) antibody (Biolegend, San Diego, CA), and CD71^+^ erythroblasts were purified using anti-biotin microbeads and a MACS system (Miltenyi Biotec GmbH).

### Genomic DNA Sequencing Analysis

The genomic DNA fragment of the exon 2 of the GATA-1 gene was PCR-amplified from mouse tail DNA using primers p1 (5′-CAA CCC CAG TGT TCC CAT GGA TT-3′) and p2 (5′-GGA GTG TCT GTA GGC CTC AGC TT-3′). The conditions for the PCR reaction were 95°C for 5 minutes, followed by 30 cycles of 95°C for 1 minute, 56°C for 1 minute, and 72°C for 40 seconds, and a final step of 72°C for 10 minutes. The PCR products of exon 2 were purified and subjected to automated nucleotide sequencing using aforementioned p1 and p2 primers.

### Hematological Parameters

Blood was obtained by tail vein bleeding and was collected into dipotassium-EDTA coated tube (BD Biosciences), and then analyzed on an automated blood differential counter (Cell-Dyn 3700, Abbott, IL; ref. 17). To measure the response to acute erythroid stimulation, anemia was induced with phenylhydrazine (PHZ) essentially as previously described [17] with some minor modifications. In brief, mice received an intraperitoneal injection of PHZ (40 mg/kg body weight in 100 µl of saline) on days 1 and 2, and their blood was taken for analysis using Cell-Dyn 3700 on days 0, 3, 6, 9, and 14.

### Analysis, Sorting and Cultivation of EPs

To analyze the frequency of EPs [21], at least 2.5 millions of BM cells were first stained with rat monoclonal antibodies against various lineage markers (GK1.5 for CD4; 53-6.7 for CD8α; RA3-6B2 for B220; RB6-8C5 for Gr-1; M1/70 for CD11b; and TER-119 for Ter119) and then with APC-labeled anti-rat IgG antibody, followed by staining with antibodies against various progenitor markers, including PE-labeled anti-IL-3Rα (CD123; 5B11), PE-labeled anti-IL-7Rα (CD127; A7R24), APC-Cy7-labeled anti-c-Kit (CD117; 2B8), Pacific Blue-labeled anti-Sca-1 (D7), PE-Cy7-labeld anti-CD71 (RI7217), and FITC-labeled anti-CD41 (integrin αIIb; MWReg30) antibodies. The Lin^−^IL-3Rα^−^IL-7Rα^−^c-Kit^+^Sca-1^−^CD71^+^CD41^−^ population defined as EP cells [21] was then analyzed by flow cytometry using an LSR II cytometer (BD Biosciences). To purify EPs, lineage-negative cells in the IL-7Rα^−^ population were first enriched from BM with a MACS system (Miltenyi Biotec GmbH), followed by staining with CD71, IL-3Rα, c-Kit, Sca-1, and CD41 [21], and sorted over FACSAria (BD Biosciences). Purified EPs were cultured in EPO-containing StemPro-34 medium (Invitrogen) essentially as previously described [20], except that SCF, insulin, dexamethasone and β-estradiol were not included in the culture medium.

### Colony-forming Assays

To detect CFU-E colonies, 1×10^5^ BM cells or 5×10^3^ sorted EPs were plated in each well in semisolid medium (MethoCult M3334; StemCell Technologies) according to the manufacturer’s protocol and CFU-E colonies were counted after 2 days in culture. To detect BFU-E colonies, 4×10^4^ BM cells were plated in each well in semisolid medium (MethoCult M3434) and primitive BFU-E colonies were counted after 7 days in culture.

### S Phase Cell Determination and Cell Death Analysis

To detect proliferation activity, purified EPs (1×10^5^ cells) were cultured for 24 hours in EPO-containing medium before 20 µM 5-ethynyl-2′-deoxyuridine (EdU) was added to the culture medium for another 3 hours. DNA synthesis was then measured using Click-iT EdU Alexa Fluor 647 Flow Cytometry Assay Kits (Invitrogen) according to the manufacturer’s protocol. Under the same analysis conditions, the fraction of cells that had sub-G1 amount of DNA (detected by PI staining) was taken as percentage of cells that had undergone apoptosis.

### Western Blot Analysis

Cells were lysed in lysis buffer (50 mM Tris-HCl at pH 7.4, 0.25% Na-deoxycholate, 150 mM NaCl, 1 mM EDTA, 1% NP-40) containing 1 mM PMSF, 1 µg/ml of aprotinin, and 1 µg/ml of leupeptin, and 50 µg of lysate was subjected to western blot analysis as described previously [18]. The antibodies used were directed against mouse GATA-1 (N6), pGATA-1(S310), Mcl-1, Bcl-X_L_, Bcl-2, Bim, p38, Stat5 (Santa Cruz Biotechnology), p-Akt(Ser473), Akt, p-Erk1/2, Erk1/2, phospho-Jak2 (Tyr1007/1008), Jak2, phospho-Stat5 (Tyr694) (Cell Signaling Technology), and E4bp4 (Abcam). The polyclonal anti-mouse pGATA-1(S26) antibody was raised in rabbit by Bethyl Laboratories Inc. (Montgomery, TX; ref. 18).

### Semi-quantitative RT-PCR Analysis

Total RNA was prepared from EPs (5×10^4^ cells) with the TRIzol reagent (Invitrogen). For semi-quantitative analysis, 200 ng of RNA was reverse transcribed into cDNA with random hexamers and SuperScript III reverse transcriptase (Invitrogen) in 20 µl of reaction volume. Level of gene expression was analyzed by amplifying 3 µl of reverse-transcribed cDNA product with primers specific for *Bcl-X_L_* (sense 5′-GCT GGG ACA CTT TTG TGG AT-3′; antisense 5′-TGT CTG GTC ACT TCC GAC TG-3′), *Bim* (sense 5′-CGA CAG TCT CAG GAG GAA CC-3′; antisense 5′-CCT TCT CCA TAC CAG ACG GA-3′), *p21^Waf1/Cip1^* (sense 5′-CCT GGT GAT GTC CGA CCT G-3′; antisense 5′-CCA TGA GCG CAT CGC AA T C-3′), and *GAPDH* (sense 5′-CTT CAT TGA CCT CAA CTA CAT G-3′; antisense 5′-TGT CAT GGA TGA CCT TGG CCA G-3′). Conditions for cDNA PCR reaction were 95°C for 2 minutes followed by 24 or 26 cycles of 95°C for 30 seconds, 60°C for 30 seconds, 72°C for 30 seconds.

### Reporter Gene Assays

The −4.6 kb *p21^Waf1/Cip1^* promoter-luciferase reporter plasmid (−4.6 kb) containing −4542 to +117 bp of the murine *p21^Waf1/Cip1^* promoter region and the same reporter plasmid in which the consensus binding site for Sp1/KLF-like factors at −21 to −13 bp was mutated from GGGCGG to GTTTTG (−21 bp mt) [22] were kindly provided by Arthur Skoultchi. Sorted EPs, cultured in medium containing EPO for 16 hours, were transfected with the wild-type (−4.6 kb) or mutant (−21 bp mt) *p21^Waf1/Cip1^* promoter-luciferase reporter plasmid along with a control vector expressing the Relina luciferase by Microporator (MP-100). Twenty-four hours after transfection, cell lysates were prepared and analyzed for luciferase activities using luciferase assay substrates according to the manufacturer’s protocol (Promega).

### Statistical Analysis

All data are presented as mean ± SD, and were analyzed by one-way analysis of variance (ANOVA) (Tukey’s post tests).

## Results

### GATA-1 Serine^26^ Phosphorylation is an Early EPO-induced Event

**Figure 1:**
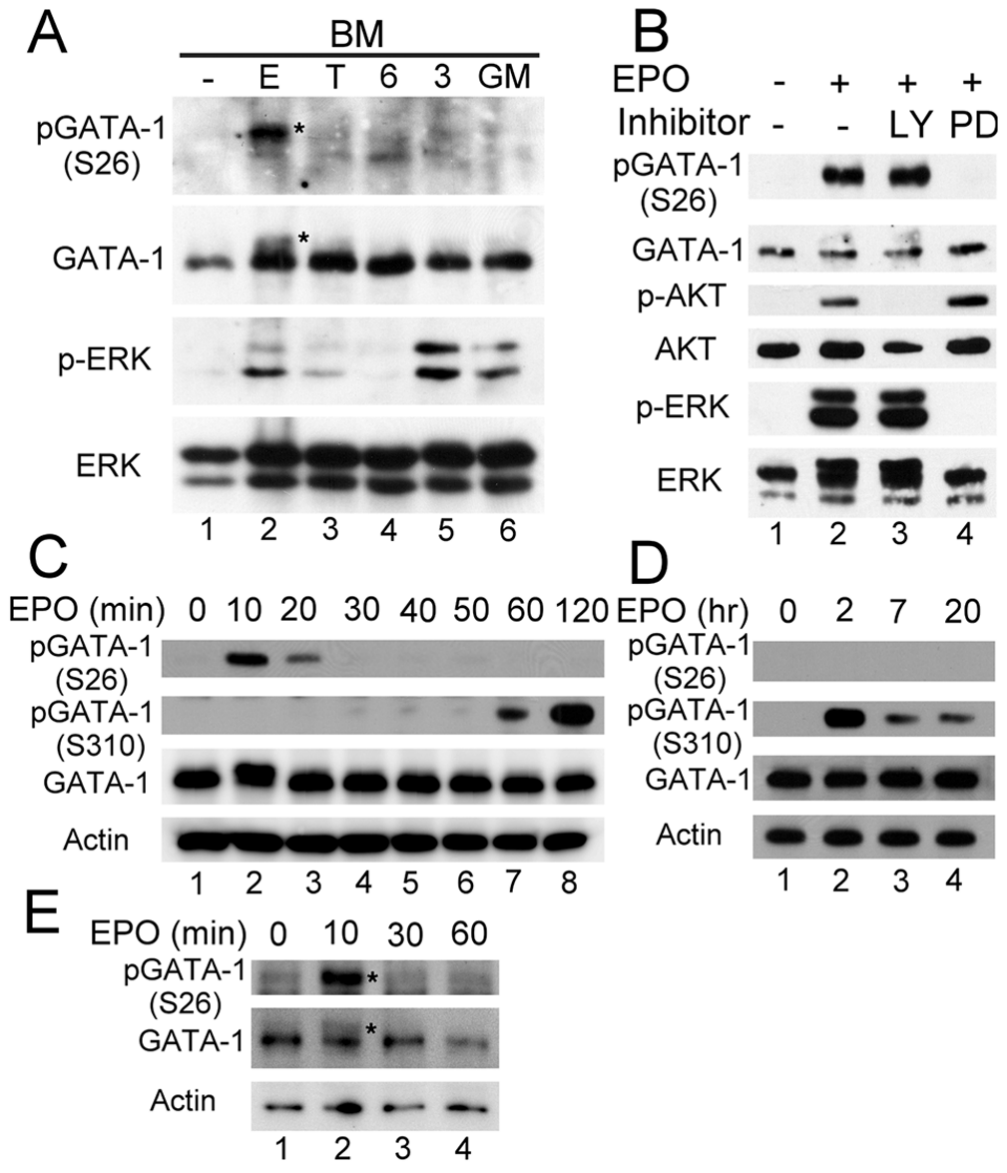
Phosphorylation of GATA-1 at serine^26^ is transiently induced by EPO in primary erythroblasts and EPs. (**A**) Fresh BM cells starved in cytokine-free RPMI 1640 medium plus 10% fetal bovine serum (FBS) for 6 hours were incubated for 10 minutes in RPMI 1640/10% FBS containing 10 U/ml of EPO (E), 50 ng/ml of TPO (T), 50 ng/ml of IL-6 (6), 50 ng/ml of IL-3 (3), or 50 ng/ml of GM-CSF (GM). Cell lysates were then analyzed by western blotting using various antibodies as indicated. pGATA-1(S26) antibody was used to detect phosphorylation of GATA-1 at serine^26^. (**B**) EPO induces GATA-1 serine^26^ phosphorylation via the MEK/MAPK-dependent pathway. Primary erythroblasts starved in cytokine-free StemPro-34 medium for 16 hours were stimulated for 10 minutes with EPO alone or together with 50 µM LY294002 (LY) or 50 µM PD98059 (PD). Total ERK and Akt were used as loading controls for the Western blotting analysis. (**C** & **D**) Sequential phosphorylation of GATA-1 at serine^26^ and serine^310^ in response to EPO stimulation. Erythroblasts were cultured and treated as in panel B, but for different time intervals as indicated. pGATA-1(S310) antibody was used to detect phosphorylation of GATA-1 at serine^310^. Expression of β-actin was used as a loading control. (**E**) Phosphorylation of GATA-1 at serine^26^ is transiently induced by EPO in EPs. Purified EPs were starved in cytokine-free StemPro-34 medium for 4 hours and stimulated with EPO for 10, 30, and 60 minutes. Lysates from equal numbers of EPs (1×10^5^ EPs per lane) were analyzed by western blotting. Asterisks indicate the slower migrating form of GATA-1 that was induced by EPO stimulation. Data shown for each panel were one representative result from 2–3 independent experiments.

Our previous study revealed that IL-3 induced marked phosphorylation of GATA-1 at serine^26^ [18]. Given that GATA-1 plays a critical role in erythropoiesis and that EPO is essential for erythroid production by preventing committed erythroid progenitors from undergoing apoptosis and allowing them to proliferate and differentiate [23], [24], [25], [26], it was of interest to investigate whether phosphorylation of GATA-1 at serine^26^ could be induced by EPO signaling in erythroid cells. To address this issue, freshly prepared BM cells were stimulated by various cytokines and the phosphorylation of ERK and GATA-1 were examined. As shown in Fig. 1A, phosphorylation of ERK1/2 could be detected in cells treated for 10 minutes with EPO, thrombopoietin (TPO), IL-3 or GM-CSF (lanes 2,3,5,6), but not with IL-6 (lane 4).Under the same conditions, serine^26^-phosphorylated GATA-1, detected by a specific anti-pGATA-1(Ser26) antibody [18] and appearing as a slow migrating form of GATA-1 (lane 2, indicated by asterisks), was almost exclusively found in EPO-treated cells. Importantly, phosphorylation of GATA-1 at serine^26^ was also prominently detected in cultured primary erythroblasts [20] or EPs (Lin^−^IL-3Rα^−^IL-7Rα^−^c-Kit^+^Sca-1^−^CD71^+^CD41^−^) [21] following EPO stimulation (Fig. 1B, lane 2, and Fig. 1E below). Consistent with our previous findings [18], EPO-induced phosphorylation of GATA-1 at serine^26^ was inhibited by the MEK inhibitor PD98059 (Fig. 1B, lane 4), but not by the PI-3K inhibitor LY294002 (lane 3), suggesting that EPO induces phosphorylation of GATA-1 at serine^26^ in erythroid cells via a MAPK-dependent signaling pathway.

Serine^310^ phosphorylation of GATA-1 in response to EPO stimulation has been previously reported in the HCD57 murine erytholeukemia cell line and mouse fetal liver cells [15]. We thus compared the phosphorylation kinetics of GATA-1 at serine^26^ and serine^310^ in primary erythroblast cultures in response to EPO treatment. As shown in Fig. 1C, phosphorylation of GATA-1 serine^26^ was rather transient, which peaked at 10 minutes and disappeared by 30 minutes after EPO treatment. In contrast, Akt-dependent phosphorylation at serine^310^ of GATA-1 [15] was not prominent until 1–2 hour of EPO treatment (Fig. 1C), and such phosphorylation event remained detectable even 7 to 20 hours after stimulation (Fig. 1D, lanes 3 and 4). These results indicate that phosphorylation of GATA-1 at serine^26^ in primary erythroblasts is an early, but transient, event following EPO stimulation. Notably, EPO-induced transient phosphorylation of GATA-1 at serine^26^ in EPs also manifested a similar kinetics as that observed in primary erythroblasts (Fig. 1E).

### Generation of GATA-1^S26E^ and GATA-1^S26A^ mice

**Figure 2:**
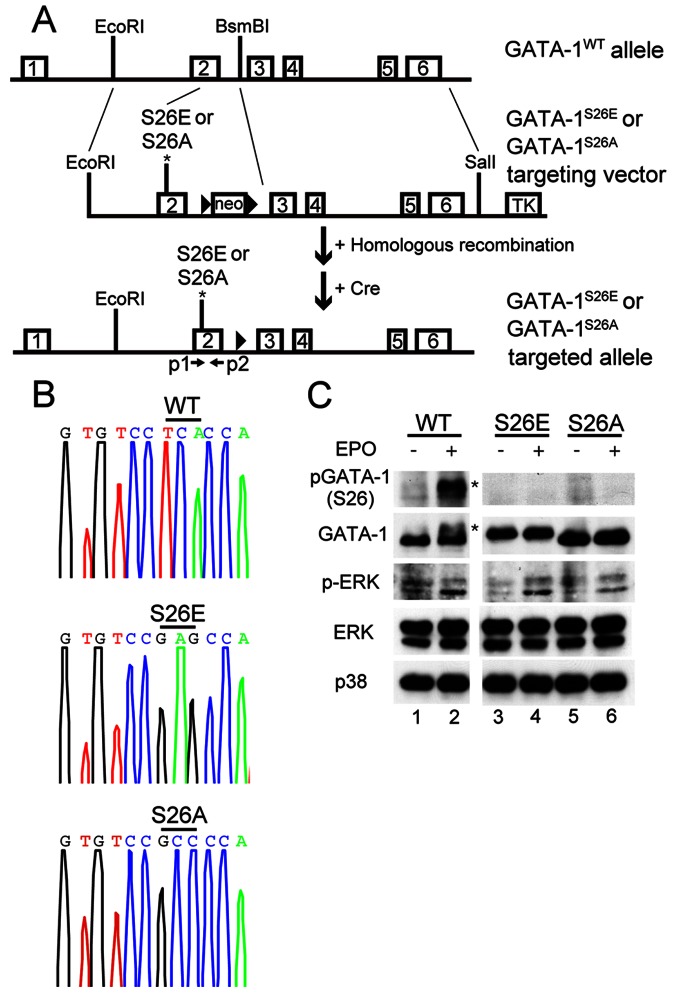
Generation of GATA-1^S26E^ and GATA-1^S26A^ mutant mice. (**A**) Schematic representation of the GATA-1^S26E^ and GATA-1^S26A^ gene-targeting strategy. The numbered boxes are exons of the GATA-1 gene. Point mutation of serine^26^ to glutamic acid or alanine in exon 2 is indicated by an asterisk (*) and relevant restriction sites are as depicted. The solid triangles represent loxP sites. PCR primers (p1 and p2) used to sequence exon 2 (see below) of the GATA-1 gene are as indicated. (**B**) Confirmation of point mutations by DNA sequencing. Tail DNA from GATA-1^S26E^ and GATA-1^S26A^ hemizygous male mice were amplified with primers p1 and p2 and the PCR products were analyzed by the automatic sequencer. The “WT” allele shows the TCA codon for serine; the “S26E” allele shows the GAG codon for glutamic acid, and the “S26A” allele shows the GCC codon for alanine. (**C**) GATA-1^S26E^ and GATA-1^S26A^ proteins are resistant to EPO-induced phosphorylation. Primary erythroblasts purified from GATA-1^S26E^ and GATA-1^S26A^ mutant mice were starved for 16 hours and stimulated with EPO for 10 minutes. Cell lysates were subsequently analyzed using various antibodies as indicated.

To explore the physiological significance of GATA-1 phosphorylation at serine^26^ on erythropoiesis *in vivo*, two knock-in mice bearing a specific amino acid replacement at serine^26^, one to glutamic acid (GATA-1^S26E^) and the other to alanine (GATA-1^S26A^; Fig. 2A), were generated. The glutamic acid substitution was expected to create a mutant protein that would mimic the phosphorylated form of GATA-1, whereas the alanine substitution would generate a GATA-1 mutant that can not be phosphorylated at serine^26^ [18]. The introduction of gene mutation at serine^26^ in mice was verified by sequencing of the genomic DNA from knock-in mice (Fig. 2B). Both GATA-1^S26E^ and GATA-1^S26A^ hemizygous males were born with an expected Mendelian frequency. The adult mice were fertile and morphologically normal, and survived for nearly two years without any obvious defect. BM levels of endogenous GATA-1^S26E^ and GATA-1^S26A^ proteins in the mutant mice appeared to be very similar to those in the wild-type mice (Fig. 2C), and, as expected, these proteins were refractory to phosphorylation after 10 minutes incubation with EPO (Fig. 2C). Notably, GATA-1^S26E^ appeared as a slowly migrating form (Fig. 2C, compare lanes 3&4 to lanes 5&6), which is consistent with our previous results obtained from a transient expression study using the Ba/F3 cell line [18].

### Normal Peripheral Blood Compositions in GATA-1^S26E^ and GATA-1^S26A^ mice

**Figure 3:**
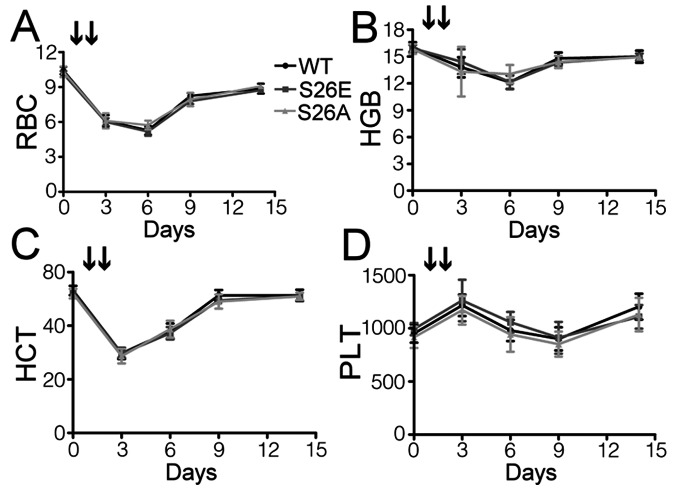
The response to hemolytic anemic stress is unchanged in mutant mice. Hemolytic anemia was induced by two consecutive injections of PHZ (indicated by arrows) as described in “*Materials and Methods*”. Red blood cell count (RBC: ×10^9^ cells/mL, **A**), hemoglobin (HGB: g/dL, **B**), hematocrit (HCT: %, **C**), and platelets (PLT: ×10^6^ cells/mL, **D**) in the peripheral blood were measured at the indicated time points during recovery from anemia. All data are shown as mean ± S.D. (n = 9 for each genotype).

To examine whether overall hematopoiesis was affected in GATA-1^S26E^ and GATA-1^S26A^ mice, the peripheral blood composition of control and both mutant mice were analyzed. As shown in Table 1, all parameters examined (e.g., white blood cells, red blood cells, platelets, hemoglobin, hematocrit, mean corpuscular hemoglobin concentration, etc) were comparable between control and either mutant mice, suggesting that phosphorylation or dephosphorylation of GATA-1 at Ser^26^ does not significantly affect the steady-state hematopoiesis of the animals. We next examined whether erythropoiesis was affected in the mutant mice under stress conditions. To address this issue, acute hemolytic anemia was induced by injection of PHZ on two consecutive days. As shown in Fig. 3, the time course and magnitude of the changes induced by PHZ treatment in the number of red blood cells, percentage of hematocrit, hemoglobin concentration, and platelet number were all comparable in the three genotypes. These results indicate that GATA-1 phosphorylation or dephosphorylation at serine^26^ does not significantly impair the ability of the mice to recover from anemic stresses.

**Table 1:** Hematological parameters of whole blood in GATA-1^S26E^ and GATA-1^S26A^ mice.

	WT	GATA-1^S26E^	GATA-1^S26A^
WBCs (10^6^/ml)	12.7±2.6	11.8±2.5	11.5±2.5
Eosinophils (10^6^/ml)	0.25±0.2	0.29±0.1	0.31±0.2
RBCs (10^9^/ml)	10.2±0.3	10.1±0.4	10.4±0.2
HGB (g/dl)	15.8±0.4	15.9±0.5	15.9±0.4
HCT (%)	52.2±1.6	52.7±1.9	52.1±1.3
MCV (fl)	50.8±0.9	51.9±0.9	49.8±0.9
MCHC (g/dl)	30.3±0.4	30.3±0.3	30.4±0.3
Platelets (10^6^/ml)	889±81	909±99	887±81
MPV (fl)	6.1±0.4	6.4±0.4	5.9±0.4

The blood was collected from 8- to 9-weeks old male mice with the indicated genotypes. All data are shown as mean ± SD (n = 16–37). WBCs, white blood cells; RBCs, red blood cells; HGB, hemoglobin; HCT, hematocrit; MCV, mean corpuscular volume; MCHC, mean corpuscular hemoglobin concentration; MPV, mean platelet volume.

**Figure 4:**
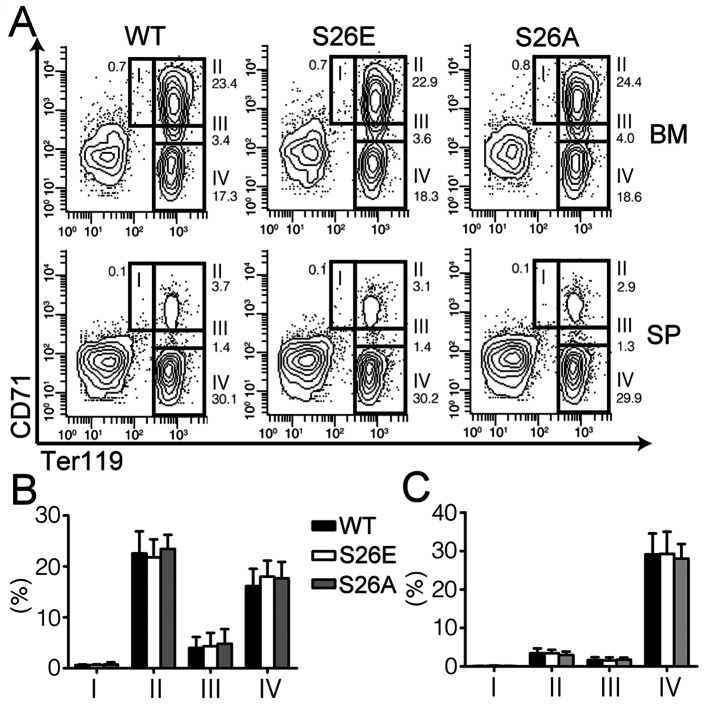
Lack of effect of serine^26^ mutations on the development of erythroblasts. (**A**) Representative FACS profiles of the erythroblast population in adult BM and spleen (SP) from wild-type (WT), GATA-1^S26E^ (S26E), and GATA-1^S26A^ (S26A) male mice. Four erythroblast stages (I–IV) were defined by the combined differential expression of CD71 and Ter119 as follows: stage I, proerythroblasts (CD71^high^/Ter119^med^); stage II, basophilic erythroblasts (CD71^high^/Ter119^high^); stage III, late basophilic and polychromatophilic erythroblasts (CD71^med^/Ter119^high^); and stage IV, orthochromatic erythroblasts (CD71^low^/Ter119^high^). The percentages of erythroblast at each stage as indicated in (**A**) are shown in (**B**) for the BM and (**C**) for the spleen. All data are shown as mean ± SD (n = 9–10 for each genotype). The P values for all four stages among the three genotypes are >0.1.

The hematopoietic system is known to have remarkable feedback systems that would compensate for abnormalities in the BM, and maintain normal peripheral blood parameters [27], [28]. We thus explored the possibility that earlier stages of erythropoiesis might be affected in these mutant mice. We first examined whether erythroid differentiation in BM or the spleen was affected by using FACS-based profiling of erythroblast subpopulations using CD71 and Ter119 markers [29]. The four erythroblast subpopulations with increasing maturity (I–IV) were defined as follows: stage I, proerythroblasts (CD71^high^/Ter119^med^); stage II, basophilic erythroblasts (CD71^high^/Ter119^high^); stage III, late basophilic and polychromatophilic erythroblasts (CD71^med^/Ter119^high^); and stage IV, orthochromatic erythroblasts (CD71^low^/Ter119^high^) [29]. The results shown in Fig. 4 indicate that the proportion of cells in each subpopulation in either BM (Fig. 4A and 4B) or the spleen (Fig. 4A and 4C) of GATA-1^S26E^ or GATA-1^S26A^ mice manifested no significant differences from that in the respective organ of the control mice.

**Figure 5:**
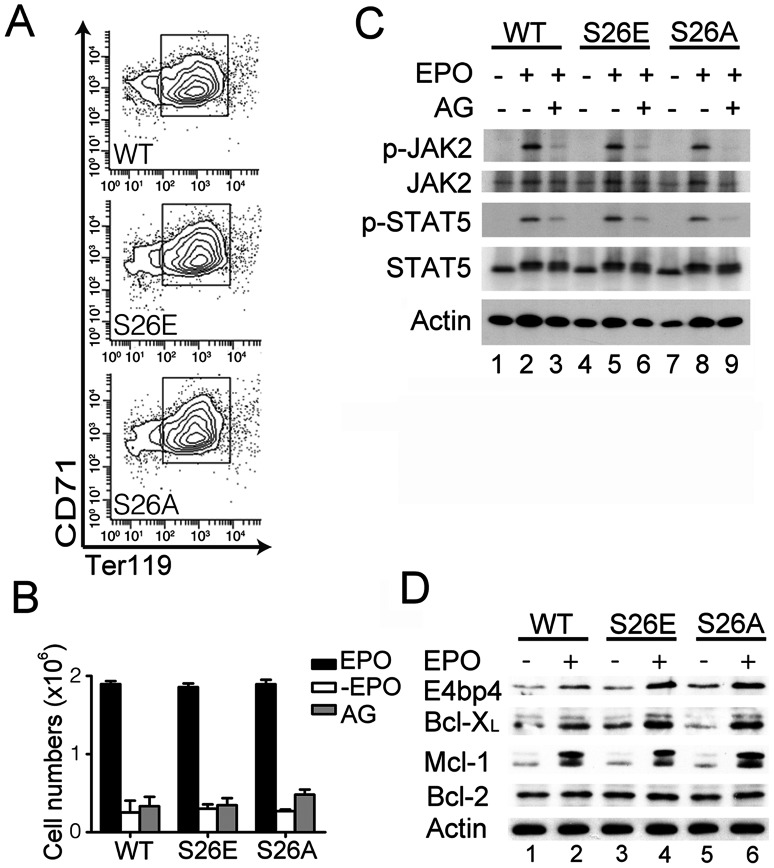
The serine^26^ mutation does not affect EPO-dependent growth or survival protein expression in primary erythroblasts. (**A**) CD71 and Ter119 expression profiles of cultured erythroblasts. Primary erythroblasts were cultured in the presence of EPO for 4 days, and purified with anti-CD71 antibody (see “*Materials and Methods*”). After purification, each erythroblast population was analyzed for CD71 and Ter119 expression. Ninety percent or more of the cells were CD71^+^ with the majority being at the stage I and II (boxed areas, see also Figure 4A). (**B**) EPO- and JAK-dependent growth of primary erythroblasts. Purified erythroblasts (1×10^6^ cells) were cultured in medium with or without EPO or with EPO plus 25 µM AG490 (AG). Twenty-four hours after culturing, total viable cell numbers were determined. (Three mice were used for each genotype and for each treatment). (**C**) AG490 inhibits EPO-induced phosphorylation of JAK2 and its downstream target STAT5. Purified erythroblasts were starved in cytokine-free medium containing or not containing AG490 for 16 hours, and then stimulated with or without EPO for 10 min prior to being lysed for Western blotting analysis using various antibodies as indicated. (D) EPO-dependent induction of survival proteins in primary erythroblasts. Purified erythroblasts were starved in cytokine-free medium for 16 hours, and then stimulated with EPO for 6 hours prior to being lysed for Western blotting analysis using various antibodies as indicated. One representative data set was shown from 3 independent experiments.

GATA-1 deficient erythroblasts die at the proerythroblast-like stage [19], [30] and our earlier study indicates that phosphorylation of GATA-1 at serine^26^ promotes Bcl-X_L_ expression and cell survival [18]. The apparent normal differentiation of erythroblasts in both mutant mice suggests that phosphorylation or dephosphorylation of GATA-1 at serine^26^ does not affect the growth or survival of erythroblasts. We thus examined this issue by using cultured primary erythroblasts which were found to be largely at stages I and II of their differentiation process (Fig. 5A). As shown in supplemental Fig. S1, the growth and death responses of these cultured primary erythroblasts in the presence or absence of EPO were very similar between cells from control or either mutant mice. Moreover, like wt cells, both mutant erythroblasts were highly dependent on EPO and the JAK activity for survival, as withdrawal of EPO or treatment with the JAK inhibitor AG490 dramatically reduced their viability (Fig. 5B and 5C). Furthermore, the extent of EPO-induced up-regulation of the anti-apoptotic proteins Mcl-1, Bcl-X_L_ and E4bp4 was very similar in cells from all three genotypes (Fig. 5D). Taken together, these results indicate that phosphorylation or dephosphorylation of GATA-1 at serine^26^ is dispensable for EPO-dependent growth and survival of cultured primary erythroblasts.

### Reduced CFU-E in GATA-1^S26E^ Bone Marrows

**Figure 6:**
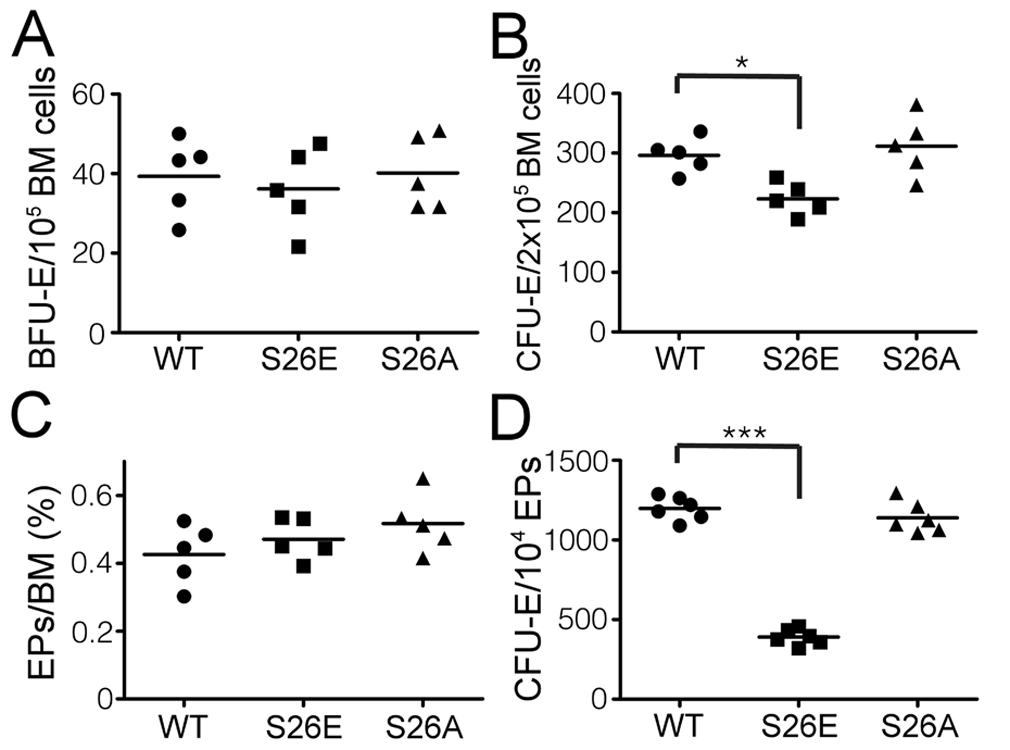
Attenuated colony forming activity of EPs in GATA-1^S26E^ mice. The frequencies of BFU-E (**A**) and CFU-E (**B**) colonies in the BM from mice with the indicated genotype were determined as described in “*Materials and Methods”*. Each dot represents the colony number for that particular mouse with the indicated genotype done in triplicate (**A**) or duplicate (**B**) and the bars indicate averages of each genotype (n = 5). (**C**) The S26E mutation does not affect the EP population frequency within the BM. The frequency of the EP population (Lin^−^IL-3Rα^−^IL-7Rα^−^c-Kit^+^Sca-1^−^CD71^+^CD41^−^) in the BM of mice with the indicated genotype was analyzed by flow cytometry. (**D**) The CFU-E activities of freshly-isolated EPs were determined as described in “*Materials and Methods”* and the results are graphically presented as that described in the legends to panels **A** & **B**. *, P<0.05; ***, P<0.001.

The erythroblast differentiation appeared to be normal in both GATA-1^S26E^ and GATA-1^S26A^ mice. However, this does not exclude the possibility that some defects occur in the BM, but are somehow compensated at later stages of erythropoiesis. We thus examined whether the progenitor population in the BM of these mutant mice was affected. To address this issue, colony forming assay was performed. Fig. 6A shows that BM cells from mice of all three genotypes contained comparable number of BFU-E progenitors and most BFU-E colonies formed from these three different groups were very similar in size and morphology (Fig. S2A). In contrast, a significant reduction (about 25% compared to wild-type) in the CFU-E progenitor population was found in the BM from GATA-1^S26E^, but not from GATA-1^S26A^ mice (Fig. 6B), albeit most CFU-E colonies formed from the GATA-1^S26E^ BM cells were also very similar in size and morphology to those formed from the BM cells of wt or GATA-1^S26A^ mice (Fig. S2A). Next, since the EP population (Lin^−^IL-3Rα^−^IL-7Rα^−^c-Kit^+^Sca-1^−^CD71^+^CD41^−^) was shown to account for most CFU-E activity in the bone marrow [21], we thus examined whether this population of cells was affected in GATA-1^S26E^ mice by flow cytometry (Fig. S2B, and Fig. 6C). Surprisingly, the result showed that the frequency of EP population in the BM of GATA-1^S26E^ mice was actually comparable to that in wild-type or GATA-1^S26A^ mice (Fig. 6C). Such unexpected result prompted us to compare the CFU-E forming activity of purified EPs from mice of all three genotypes. Fig. 6D shows that, compared to controls, a significantly lower number of CFU-Es was generated from GATA-1^S26E^ but not from GATA-1^S26A^ EPs, suggesting that the reduced CFU-E forming activity observed in GATA-1^S26E^ bone marrow is due to an intrinsic defect in the EP population.

**Figure 7:**
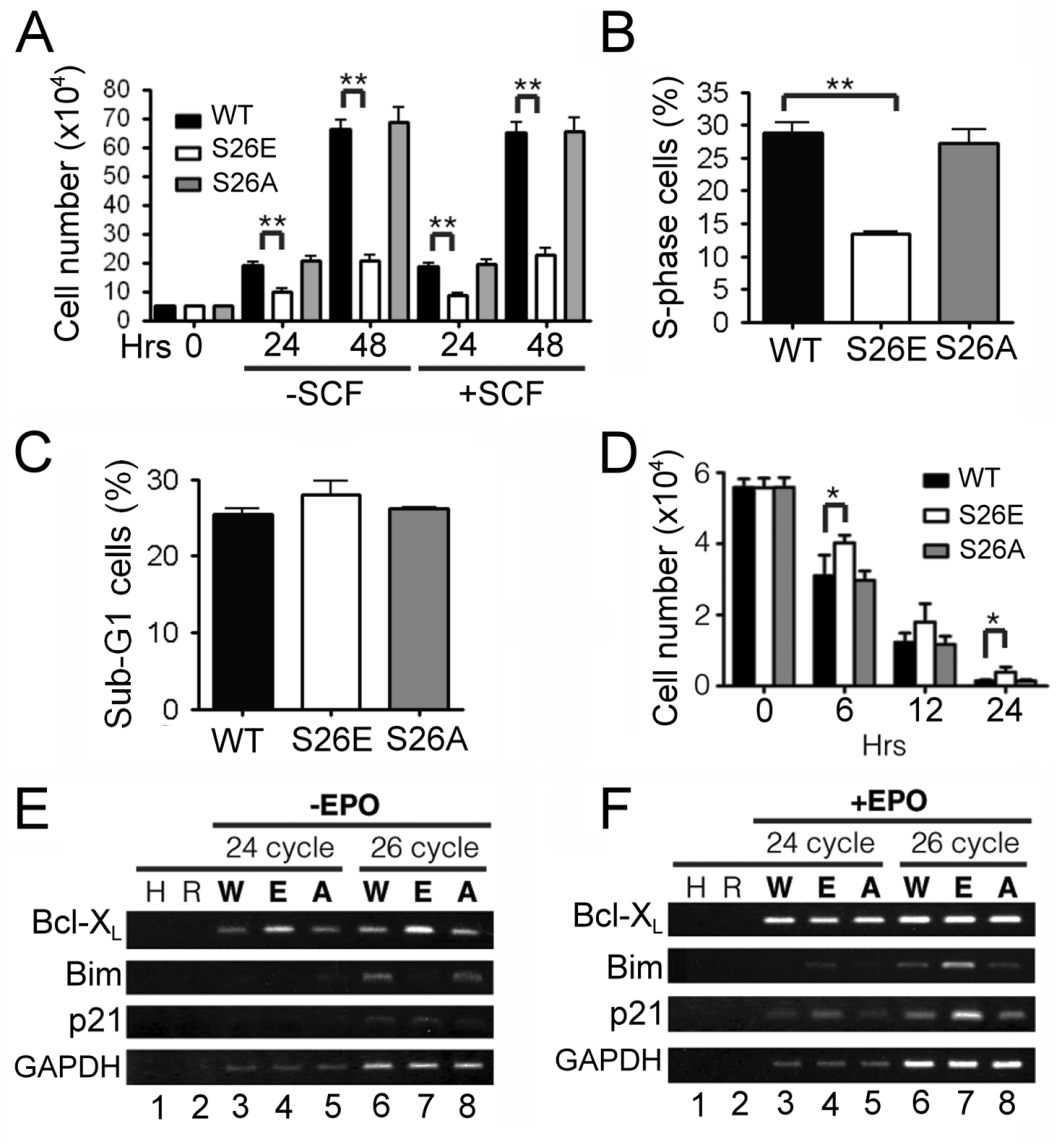
Differential responses of GATA-1^S26E^ EPs in medium with or without EPO. (**A-C**) Impaired growth of GATA-1^S26E^ EPs in medium containing EPO. (**A**) Purified EP cells with the indicated genotype were cultured in medium containing EPO plus the presence or absence of SCF and the viable cell numbers at various time points were determined. (**B,C**) Same as in (**A**) in culture without SCF except that the percentage of cells in S phase (**B**) or with sub-G1 amount of DNA (**C)** after 24 hr’s culturing was analyzed by flow cytometry (see *Materials and Methods*). (See also Figure S3 for one representative contour plot of the flow cytometric analysis shown in panels **B** and **C**). (**D**) Survival advantage of GATA-1^S26E^ EPs in medium without EPO. Purified EP cells were cultured in medium without EPO and viable cell numbers at various time points were determined. All results shown in panels **A-D** are mean ± SD (n = 3–6 for each genotype). (**E**) Analysis of mRNA expression of genes related to cell growth and/or survival by RT-PCR. Purified EPs with the indicated genotype were cultured in medium without (**E**) or with (**F**) EPO for 6 hours, and mRNA expression levels of the indicated genes were analyzed by semi-quantitative RT-PCR analysis (24 or 26 cycles). H and R are negative controls using water or without RT, respectively, in the analysis buffer. W,E and A denote RNA from control, GATA-1^S26E^ and GATA-1^S26EA^ EPs, respectively. P21, p21^Waf1/Cip1^. Both (**E**) and (**F**) are one representative data set from 3 independent experiments with very similar results (see Figure S4 for quantification of these results). *, P<0.05; **, P<0.01.

We next employed a regular suspension cell culture system to further dissect the colony forming defect of GATA-1^S26E^ EPs. As shown in Fig. 7A, total cell number of wild-type or GATA-1^S26A^ EP was increased for nearly 4 fold after the culture with EPO was initiated for 24 hours. However, under the same experimental conditions, substantially fewer viable cells were observed in culture using purified GATA-1^S26E^ EPs compared to those using wild-type or GATA-1^S26A^ EPs. Further addition of SCF to such culture system still failed to rescue the growth defect of GATA-1^S26E^ EPs (Fig. 7A), excluding the possibility that the observed defect in the CFU-E but not BFU-E activity of GATA-1^S26E^ BM cells was due to lack of SCF in the former assay system. We next examined whether reduction in the viable cell number in the GATA-1^S26E^ EP culture was due to defects in cell proliferation or survival or both. To address this issue, purified EPs were cultured in EPO-containing medium for 24 hrs, pulse-labeled with the thymidine analog 5-ethynyl-2′-deoxyuridine (EdU) for additional 3 hours and the percentage of cells in S phase or with sub-G1 amount of DNA (apoptotic cells) was analyzed by flow cytometry. As shown in Fig. 7B, 7C and Fig. S3, compared to wt or GATA-1^S26A^ cells, GATA-1^S26E^ EPs manifested a significant reduction of cells in the DNA synthesis (S) phase (∼50% of wt level, Fig. 7B), but only a marginal increase of cells that had undergone apoptosis (Fig. 7C). Together, these results suggest that reduced colony forming activity of GATA-1^S26E^ EPs in EPO-containing medium might be largely due to decreased cell proliferation of these cells.

Of note, although the survival of GATA-1^S26E^ EPs in EPO-containing medium was only marginally reduced compared to that of control cells, such result was somewhat surprising, because our earlier study indicated that ectopic expression of GATA-1^S26E^ suppressed IL-3-withdrawal-induced apoptosis [18]. We thus compared the survival response of EPs from all three genotypes in medium without EPO. Consistent with our earlier findings in Ba/F3 cells [18], GATA-1^S26E^ EPs survived better than wild-type and GATA-1^S26A^ EPs in the absence of EPO (Fig. 7D). Semi-quantitative RT-PCR analysis further revealed that, under such culture conditions, GATA-1^S26E^ EPs expressed higher levels of Bcl-X_L_ and a slightly lower level of pro-apoptotic Bim mRNA compared to cells from the other two genotypes (Fig. 7E, and Fig. S4A). In contrast, a quite different result was observed when these cells were cultured in medium containing EPO (Fig. 7F and Fig. S4B). First, Bcl-X_L_ expression in GATA-1^S26E^ EPs was not significantly different from that in control or GATA-1^S26A^ cells. Second, the expression of Bim was actually slightly higher in GATA-1^S26E^ EPs than in wt or GATA-1^S26A^ cells. Moreover, compared to control or GATA-1^S26A^ cells, GATA-1^S26E^ EPs expressed higher levels of the CDK inhibitor p21^Waf1/Cip1^ only in the presence but not in the absence of EPO (Fig. 7E v.s. 7F).

### Reduced CFU-E in GATA-1^S26E^ BMs was Mainly due to GATA-1^S26E^-mediated Transcriptional Activation of the p21^Waf1/Cip1^ Gene

**Figure 8:**
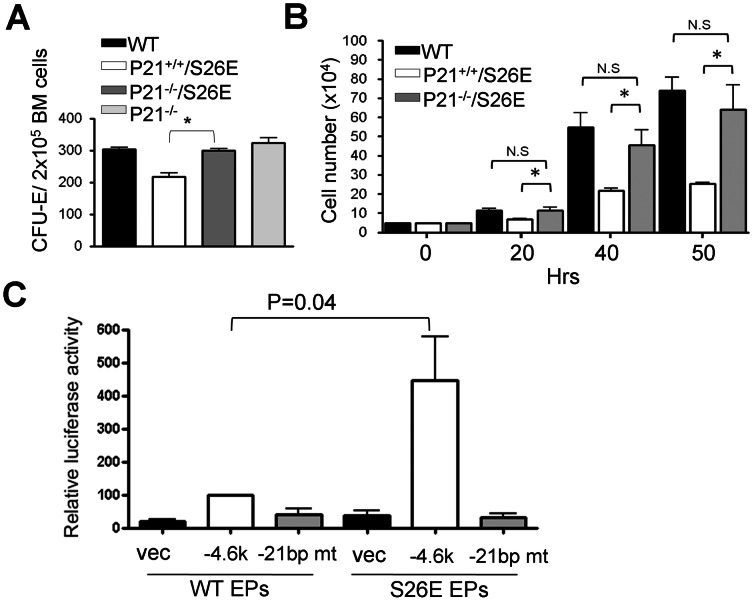
Ablation of p21^Waf1/Cip1^ restores the CFU-E number of GATA-1^S26E^ BM. (**A**) The frequency of CFU-E colony in the BM from mice with the indicated genotype was determined as that described in the legend to Figure 6B. (**B**) Purified EP cells from mice with the indicated genotype were cultured in EPO-containing medium and viable cell number was counted at indicated time points by the trypan blue exclusion assay. The numbers represent mean ± SD from 3 independent experiments for each genotype. (**C**) Luciferase reporter assays were performed in wt or GATA-1^S26E^ EPs in EPO-containing medium with promoter-less control vector (vec), wt (−4.6 K) or mutant (−21 bp mt) p21^Waf1/Cip1^ promoter-reporters as indicated. All results shown in **A-C** are presented as mean ± SD. (n = 3–5 for each genotype). *, P<0.05; n.s., P>0.05.

Next, we examined whether up-regulated expression of Bim and/or p21^Waf1/Cip1^ may account for the reduced CFU-E activity in the BM of GATA-1^S26E^ mice. To address this issue, compound mutant mice carrying the *GATA-1^S26E^* allele and the knockout allele of the *Bim* or the *p21^Waf1/Cip1^* gene were generated. Whereas ablation of Bim did not significantly revert the CFU-E numbers of GATA-1^S26E^ BM (data not shown), ablation of p21^Waf1/Cip1^ completely rescued the colony forming defect of GATA-1^S26E^ BM albeit knockout of p21^Waf1/Cip1^ itself did not affect the CFU-E activity of the mouse BM (Fig. 8A). Consistent with this result, the *in vitro* culture experiment also showed that ablation of p21^Waf1/Cip1^ restored the growth of GATA-1^S26E^ EPs in EPO-containing medium to an extent that was very similar to that of WT cells (Fig. 8B). Further reporter gene analysis revealed that, in the presence of EPO, the *p21^Waf1/Cip1^* gene promoter was significantly activated in GATA-1^S26E^ EPs (∼10-fold, compared to promoter-less vector control), but was only marginally activated in WT cells (∼3-fold, Fig. 8C). We next examined whether the consensus binding site for the Sp1/KLF-like factor at -21 to -13 bp of the *p21^Waf1/Cip1^* gene promoter was important for GATA-1^S26E^-mediated transcriptional activation of the *p21^Waf1/Cip1^* gene, as such site was shown to be critical to GATA-1-stimulated transcription of the *p21^Waf1/Cip1^* promoter [22]. The result shown in Fig. 8C indicated that GATA-1^S26E^-mediated transcriptional activation was abolished if the consensus binding site for the Sp1/KLF-like factor at −21 to −13 bp of the *p21^Waf1/Cip1^* gene promoter was mutated (−21 bp mt). Together, these results suggest that the S26E mutation confers on GATA-1 a prominent activity to activate the transcription of the *p21^Waf1/Cip1^* gene in EPs.

## Discussion

In this study, we demonstrate that phosphorylation of GATA-1 at serine^26^ is transiently induced by EPO in both erythroblasts and EPs. Mutation analysis indicates that such a transient event, i.e., quick phosphorylation followed by dephosphorylation of GATA-1 at serine^26^, is not essential for EPO-dependent growth or survival of erythroblasts. In contrast, the same mutation study revealed a different effect on EPs. While phosphorylation of GATA-1 at serine^26^ is dispensable for the proliferation and survival of EPs, failure to dephosphorylate serine^26^ following its transient phosphorylation significantly attenuates such two activities. These results suggest that constitutive phosphorylation of GATA-1at serine^26^ interferes with the development of the erythroid lineage in a stage-specific manner. We previously showed that GATA-1 phosphorylation at serine^26^ plays an important role in the survival and expression of Bcl-X_L_ and E4bp4 in IL-3-dependent Ba/F3 cells [18]. However, the expression of these proteins was not affected by the GATA-1 serine^26^ mutations in cultured primary erythroblasts, suggesting that serine^26^ phsophorylation affects survival protein expression in a cell-type and/or cell-context specific manner. Some transcriptional co-factors, which are sensitive to serine^26^ phosphorylation, may be present in cells like Ba/F3 and EPs. However, some co-factor(s), which are insensitive to serine^26^ phosphorylation, may be present in cells like erythroblasts. A recent study reported that EPO protects neuronal cells from apoptosis via the NF-κB signaling pathway [31]. This result further suggests that EPO may induce the expression of survival proteins and maintain the viability of erythroblasts via an alternative pathway such as the one involving NF-κB. More experiments are required to address this possibility.

p21^Waf1/Cip1^ is not essential for normal erythropoiesis, as mice deficient of this protein did not manifest any abnormal phenotype in the erythroid lineage (data not shown). One interesting finding from this study is that the reduced CFU-E activity of GATA-1^S26E^ EPs is mainly due to an increased expression of p21^Waf1/Cip1^ following EPO treatment, as ablation of p21^Waf1/Cip1^ restored the CFU-E number of GATA-1^S26E^ mice. Notably, in the absence of EPO, the p21^Waf1/Cip1^ expression was not activated in GATA-1^S26E^ EPs, suggesting that GATA-1^S26E^-mediated transcriptional activation of the *p21^Waf1/Cip1^* gene is dependent on an EPO-induced co-factor. One candidate co-factor would be the protein that recognizes the consensus binding site for the Sp1/KLF-like factor at −21 to −13 bp of the *p21^Waf1/Cip1^* gene promoter, since mutation of this site abolished GATA-1^S26E^-mediated transcriptional activation of the *p21^Waf1/Cip1^* gene in EPO-treated EPs. More experiments would be required to test this possibility.

The reduced CFU-E activity of GATA-1^S26E^ EPs appeared to be compensated *in vivo*, since GATA-1^S26E^ mice didn’t manifest any abnormal phenotype at later stage of erythroid development. This is reminiscent of the phenotype of knock-in mutant mice bearing the triple-alanine substitution of GATA-1 [17], which results in a reduction in the numbers of both BFU-E and CFU-E progenitors in the bone marrow, but does not perturb later stage of erythropoiesis. One possible explanation for this phenomenon is that CFU-E progenitors may respond to additional growth factor(s) other than EPO in the bone marrow, in which GATA-1 is not required for its signaling. More experiments would be required to test this possibility.

## Supporting Information

Figure S1 **Proliferation and cell survival were unaffected in GATA-1^S26E^ and GATA-1^S26A^ erythroblasts.** Purified erythroblasts (5×10^5^ cells) were cultured in StemPro-34 medium with (**A**) or without EPO (**B**), and viable cell number was counted at indicated time points by the trypan blue exclusion assay. The numbers represent the average for 3 independent experiments for each genotype and all P values are >0.1.
(TIF)

Figure S2 **(A) Representative morphology of CFU-E and BFU-E from WT, GATA-1^S26E^ and GATA-1^S26A^ mice. (B) Analysis of the frequency of GATA-1^S26E^ and GATA-1^S26A^ EPs in the bone marrow.** BM cells were stained with various cell surface markers as described in the “Materials and Methods” section of the text. To analyze the frequency of the EP population in the BM, the lineage^−^/IL-3Rα^−^/IL-7Rα^−^ triple negative population was first gated out, followed by gating for the hematopoietic progenitors (Lin^−^IL-3Rα^−^IL-7Rα^−^c-Kit^+^Sca-1^−^, **I**). Finally the percentage of the Lin^−^IL-3Rα^−^IL-7Rα^−^c-Kit^+^Sca-1^−^CD71^+^CD41^−^ population (**II**), defined as EPs, was analyzed. Representative contour plots are shown from 5 independent experiments. Numbers indicate the percentage of the indicated population in the BM.
(TIF)

Figure S3 **Impaired proliferation of GATA-1^S26E^ EPs in EPO-containing medium.** EPs purified from mice with the indicated genotype were cultured for 24 hours in EPO-containing medium and labeled for additional 3 hours with EdU. After EdU labeling, cells in S phase (EdU^+^) or with sub-G1 amount of DNA (detected by PI staining) were determined by flow cytometry. Data shown here are one representative set of contour plots from three independent experiments with very similar results. Numbers indicate the percentage of the indicated population.
(TIF)

Figure S4 **Relative mRNA expression levels of Bcl-X_L_, Bim and p21 as described in the legend to** **Fig. 7E and 7F** **of the text.** The intensity of each PCR product (the 26 cycle) was quantified and normalized to that of the GAPDH signal. The results (mean ± SD) are plotted as a relative level to that of the wt, which is set as 1. *, P<0.05; **, P<0.01; ***, P<0.001 (N = 3 for each genotype, one-way ANOVA).
(TIF)
