# Supplementary figures and images for: Constitutive Phosphorylation of GATA-1 at Serine26 Attenuates the Colony-Forming Activity of Erythrocyte-Committed Progenitors
Source: PLoS One. 2013 May 22;8(5):e64269. doi: 10.1371/journal.pone.0064269 (PMC3661471; doi:10.1371/journal.pone.0064269)

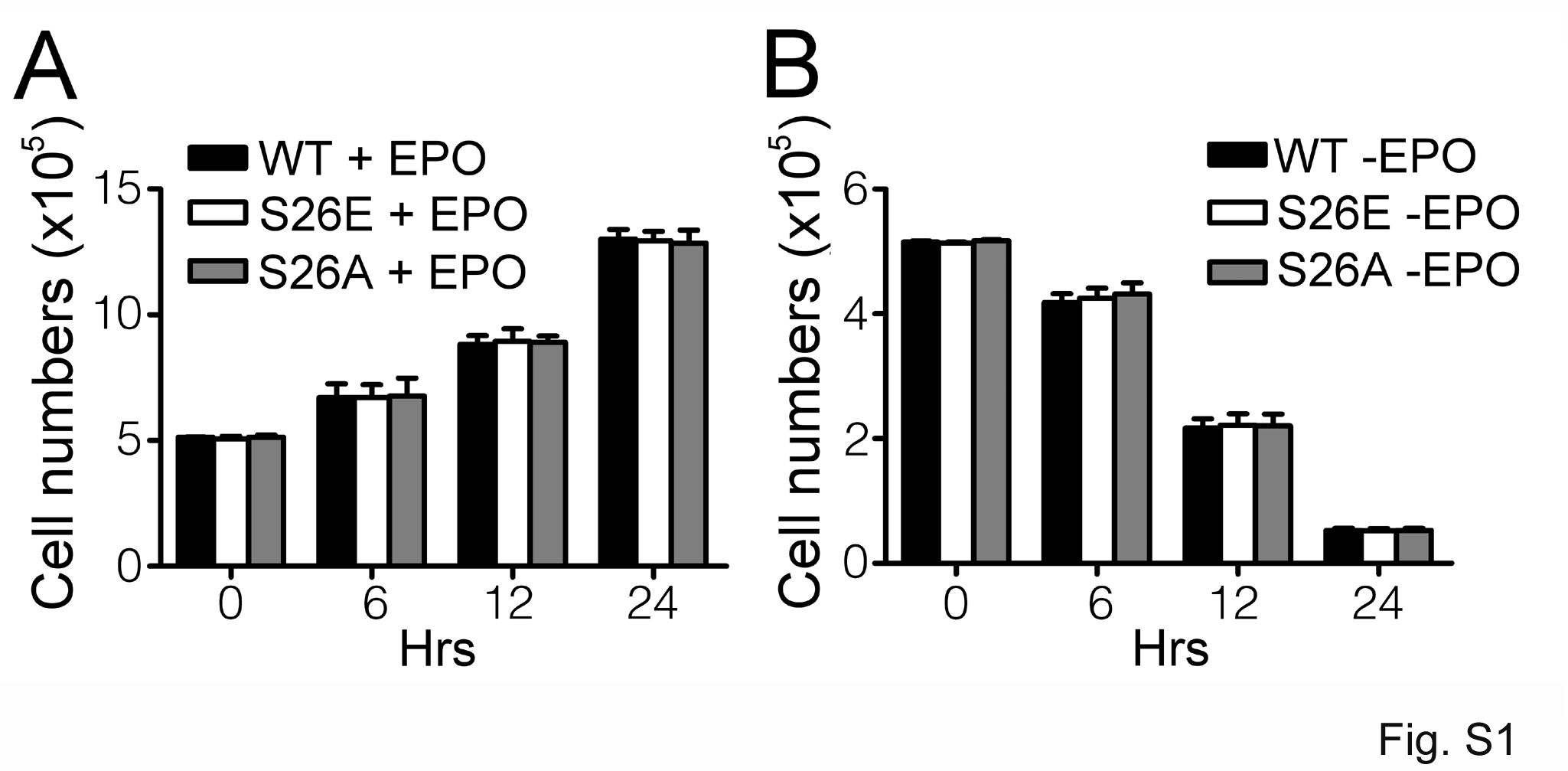

Supplement: Figure S1 — Proliferation and cell survival were unaffected in GATA-1S26E and GATA-1S26A erythroblasts. Purified erythroblasts (5×10^5^ cells) were cultured in StemPro-34 medium with (A) or without EPO (B), and viable cell number was counted at indicated time points by the trypan blue exclusion assay. The numbers represent the average for 3 independent experiments for each genotype and all P values are >0.1. (TIF) [file pone.0064269.s001.tif]

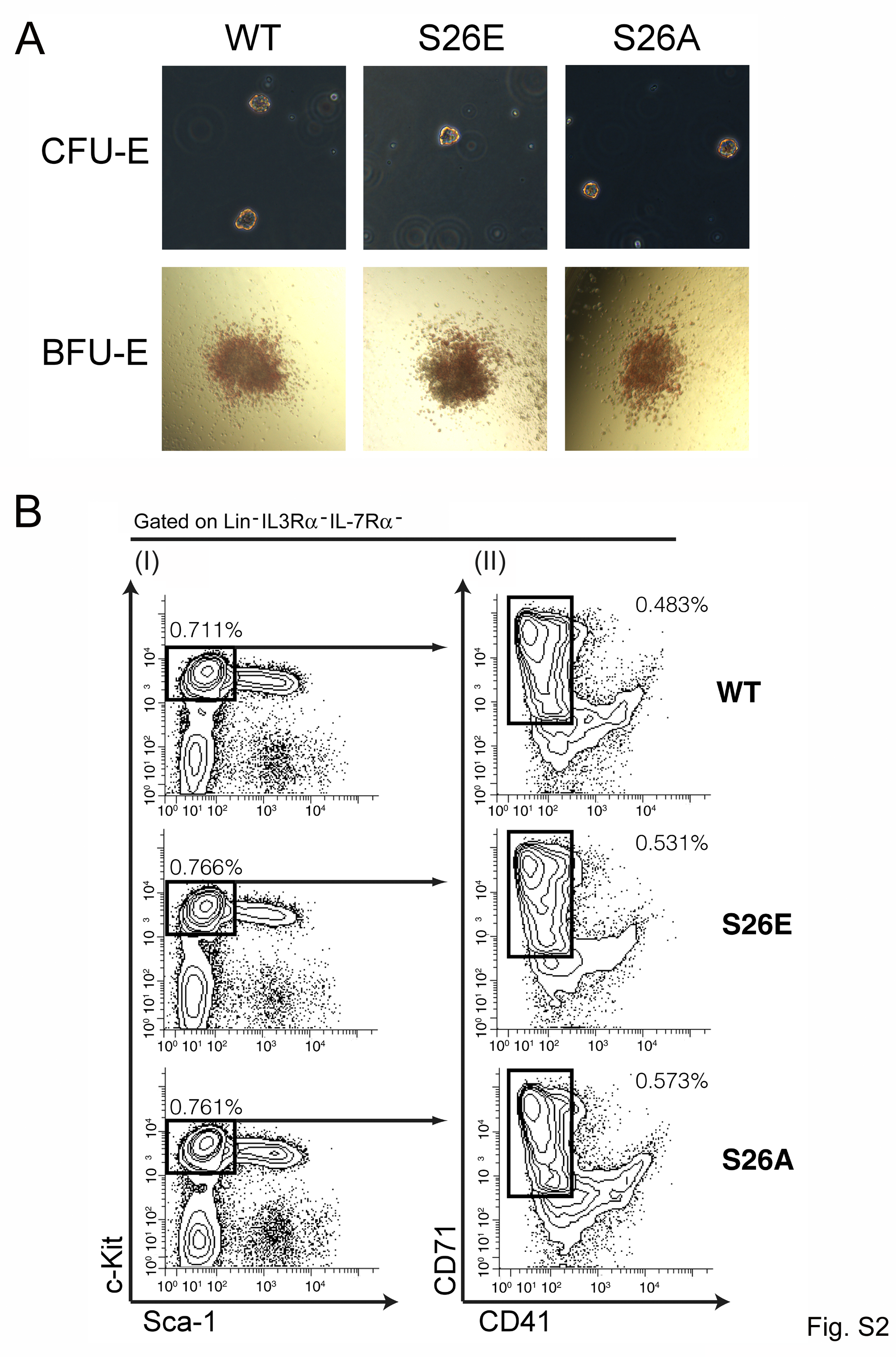

Supplement: Figure S2 — (A) Representative morphology of CFU-E and BFU-E from WT, GATA-1S26E and GATA-1S26A mice. (B) Analysis of the frequency of GATA-1S26E and GATA-1S26A EPs in the bone marrow. BM cells were stained with various cell surface markers as described in the “Materials and Methods” section of the text. To analyze the frequency of the EP population in the BM, the lineage−/IL-3Rα−/IL-7Rα− triple negative population was first gated out, followed by gating for the hematopoietic progenitors (Lin−IL-3Rα−IL-7Rα−c-Kit+Sca-1−, I). Finally the percentage of the Lin−IL-3Rα−IL-7Rα−c-Kit+Sca-1−CD71+CD41− population (II), defined as EPs, was analyzed. Representative contour plots are shown from 5 independent experiments. Numbers indicate the percentage of the indicated population in the BM. (TIF) [file pone.0064269.s002.tif]

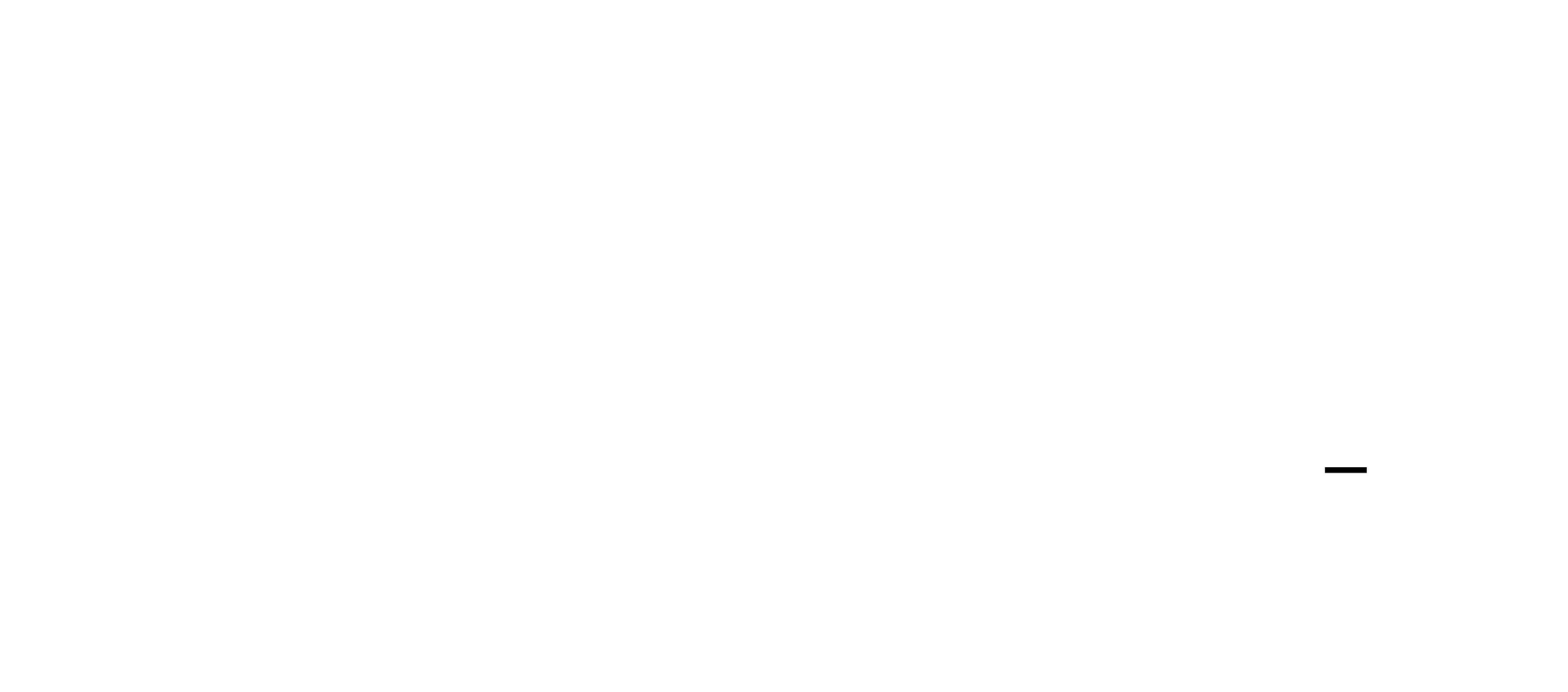

Supplement: Figure S3 — Impaired proliferation of GATA-1S26E EPs in EPO-containing medium. EPs purified from mice with the indicated genotype were cultured for 24 hours in EPO-containing medium and labeled for additional 3 hours with EdU. After EdU labeling, cells in S phase (EdU+) or with sub-G1 amount of DNA (detected by PI staining) were determined by flow cytometry. Data shown here are one representative set of contour plots from three independent experiments with very similar results. Numbers indicate the percentage of the indicated population. (TIF) [file pone.0064269.s003.tif]

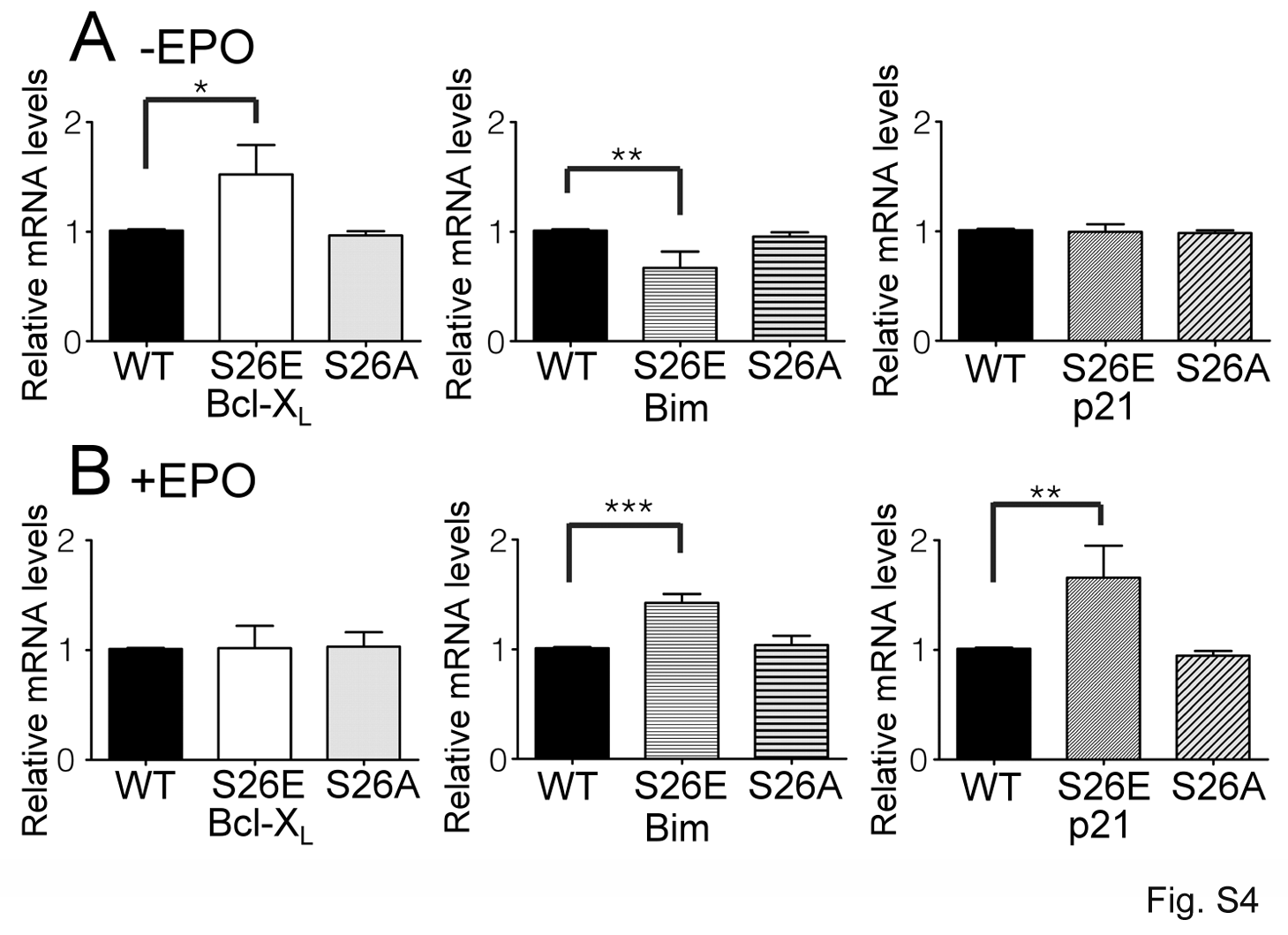

Supplement: Figure S4 — Relative mRNA expression levels of Bcl-XL, Bim and p21 as described in the legend to Fig. 7E and 7F of the text. The intensity of each PCR product (the 26 cycle) was quantified and normalized to that of the GAPDH signal. The results (mean ± SD) are plotted as a relative level to that of the wt, which is set as 1. *, P<0.05; **, P<0.01; ***, P<0.001 (N = 3 for each genotype, one-way ANOVA). (TIF) [file pone.0064269.s004.tif]
